# Effect of High Selective Serotonin-Reuptake Inhibitor Doses on the Development and Treatment of Sarcoid-Like Reaction

**DOI:** 10.1155/2020/9751837

**Published:** 2020-04-25

**Authors:** Badr Rashed Al-Ghamdi

**Affiliations:** Department of Internal Medicine, College of Medicine, King Khalid University, Abha, Saudi Arabia

## Abstract

Sarcoidosis is a systemic disorder characterized by the presence of noncaseating granulomas that are most commonly observed in the lungs. Sarcoid-like reaction has been reported to develop in response to several immune modulator agents and antidepressants. In this report, a case of pulmonary sarcoidosis that was strongly related to the use of more than the average recommended dose of selective serotonin-reuptake inhibitor (SSRI) medications has been described. The patient, a 37-year-old, single, Caucasian woman, who suffered from severe depression and who presented to the emergency department with shortness of breath, low-grade fever, a dry cough, fatigue, and the loss of appetite, was diagnosed with this condition, and she failed to respond to the administered sarcoidosis treatment until the SSRI medications that she was using were discontinued; furthermore, she relapsed when one of these medications was reintroduced. Based on these observations, we were able to show a possible relationship between sarcoid-like reaction and SSRIs, and therefore, in the case of patients with interstitial lung diseases who are using SSRIs, we recommend that meticulous precautions be taken while planning their treatment and careful follow-ups be implemented to monitor their progress.

## 1. Introduction

Sarcoidosis is a systemic disorder that preferentially affects the lungs; however, the disease is characterized by the presence of noncaseating granulomas in virtually any organ. Its pathogenesis is believed to be an antigen-specific immune response and inflammatory response to yet unidentified triggering agents. Furthermore, its clinical presentations are extremely variable and can range from an acute self-limiting illness to a severe chronic disease [1]. This disease occurs worldwide and affects people of all racial and ethnic backgrounds, of both the sexes, and of all ages, with a slight female predominance [1]. Most patients are diagnosed in adulthood prior to when they are 40 years old. However, the incidence of sarcoidosis varies widely throughout the world, which is probably because of differences in environmental exposures, surveillance methods, and predisposing human leukocyte antigen (HLA) alleles and other genetic factors [1]. The highest annual incidence of sarcoidosis, up to 40 cases per 100,000 people, has been observed in northern European countries and in the US while in Asia, the annual incidence ranges from one to two cases per 100,000 people [2]. From these data, it appears that both the Caucasian German and US population are more susceptible to sarcoidosis [1].

The American Thoracic Society, European Respiratory Society, and World Association of Sarcoidosis and Other Granulomatous Disorders (WASOG) stipulate the following criteria for the diagnosis of sarcoidosis: the presence of a consistent clinical and radiological picture, a tissue biopsy showing noncaseating granulomas, and the exclusion of other conditions that produce a similar pathology, including infections, autoimmune disorders, and inhalational diseases [3].

Additionally, drug-induced sarcoid-like reaction has been reported to occur in response to some immune-modulating agents [4–9]. However, to the best of our knowledge, there are no case reports in the literature that clearly demonstrate the association that exists between severe pulmonary sarcoidosis (PS) and the use of specific selective serotonin-reuptake inhibitors (SSRIs). Therefore, herein, we have reported a case involving PS associated with the use of more than the average recommended dose of certain SSRIs.

## 2. Case Presentation

A 37-year-old, single, Caucasian woman presented to the emergency department with a 3-month history of experiencing progressive shortness of breath that was associated with bilateral lung infiltrates. She was examined in several clinics and was treated with several courses of different oral antibiotics; however, her condition did not improve, and her shortness of breath progressively worsened over time. She reported a history of low-grade fever, a dry cough, fatigue, and the loss of appetite. Furthermore, she did not have a history of skin lesions, joint pains, or any symptoms that suggested connective tissue diseases. Additionally, her past history revealed that she had been suffering from severe depression for which she was taking oral antidepressants. Upon presentation, she was found to be on daily doses of two SSRI medications. She was prescribed paroxetine 50 mg to be taken once daily, which she was taking for the last 1 year, and owing to a misunderstanding, she was also prescribed escitalopram 10 mg to be taken once daily. As a result, she was taking both medications together for at least the last 6 months.

A physical examination disclosed that the young lady was suffering from moderate respiratory distress and peripheral cyanosis and that her oxygen saturation, measured by pulse oximetry, was 84% in the room air. During chest auscultation, she exhibited fine bilateral basal crepitations, but she did not exhibit wheezing. Furthermore, she did not present finger clubbing, skin abnormalities, or palpable lymphadenopathy. The examination of her cardiovascular system was unremarkable, and an abdominal examination did not reveal palpable organomegaly. The musculoskeletal and central nervous system examinations also did not reveal any abnormalities.

Furthermore, her initial investigations revealed a normal complete blood count (CBC), and her renal and liver function tests were normal as well. Her arterial blood gases showed type I respiratory failure with a partial pressure of oxygen (PO2) of 54 mmHg and a normal pH. The electrocardiogram (ECG) displayed sinus tachycardia with a heart rate of 125 per minute. A chest X-ray captured on presentation revealed a bilateral interstitial pattern with prominent hila that is suggestive of enlarged hilar adenopathy without pleural reactions.

**Figure 1:**
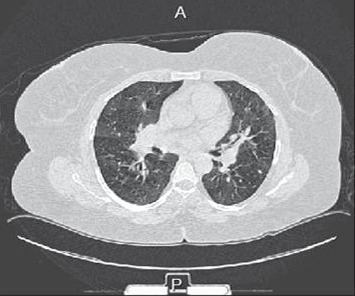
A high-resolution computed tomography (HRCT) scan of the patient chest showing a bilateral micronodular interstitial pattern with hilar adenopathy.

She was initially admitted for a chest infection; however, her condition did not improve with IV antibiotics. The results of a further work-up were consistent with those associated with a diagnosis of interstitial lung disease (ILD). A high-resolution computed tomography (HRCT) scan of her chest confirmed a bilateral micronodular interstitial pattern with hilar adenopathy (Figure 1).

**Figure 2:**
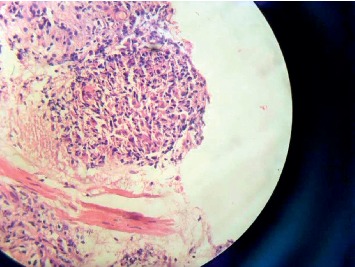
Transbronchial lung biopsy obtained from the patient showing a noncaseating granuloma.

Consequently, she required fibro-optic bronchoscopy and a transbronchial lung biopsy. The biopsy revealed a noncaseating granuloma (Figure 2).

However, the results of all the microbiological examinations, including those of the polymerase chain reaction (PCR) tests, were negative for *Mycobacterium tuberculosis*. Further, the results of all the autoimmune screening tests were also negative. Following a complete discussion, during a chest department team meeting, she was diagnosed with sarcoidosis.

Subsequently, she was started on IV methylprednisolone 60 mg BD, and due to her history of severe depression, she was orally administered escitalopram 10 mg once daily as well. Nevertheless, her respiratory status continued to deteriorate, and her oxygen requirement increased. Her condition worsened over the next few days, and she was eventually intubated and shifted to ICU for mechanical ventilation. At this stage, due to the lack of an adequate response to systemic steroids, she underwent a full re-evaluation, including another bronchoscopy (without a biopsy), by ICU team, which did not generate any new findings. Following this, broad-spectrum antibiotic coverage was added to her treatment plan for a possible nosocomial infection. However, a full septic screen failed to show any positive outcomes. A quick literature review disclosed that there is a possible correlation between granulomatous lung diseases and antidepressants [10–13]. For this reason, escitalopram was discontinued in the ICU, and the methylprednisolone dose was increased to 100 mg BD. After a few days, she started to exhibit progressive signs of improvement in her respiratory status, and she was weaned off mechanical ventilation within 12 days.

She was then shifted to the general ward where she continued to demonstrate remarkable improvement in her condition. She was subsequently examined by the psychiatry department, and it was decided that she should avoid taking SSRIs while being treated for her depression. As a result, she was started on amitriptyline orally, the IV steroids were replaced with oral prednisolone 50 mg OD, and all the antibiotics were discounted. Consequently, she continued to show progressive improvement in the general ward, and she was able to walk without an oxygen supply; furthermore, her oxygen saturation approached 90% in the room air. Within 10 days, she was discharged from the hospital. Her doses of oral steroids were kept the same, and she was given an appointment for a follow-up at the chest clinic after a month.

During her follow-up, it was observed that she continued to show progressive improvement with normal oxygen saturation and good exercise tolerance. Her oral steroid doses were gradually reduced to prednisolone 15 mg once daily to be taken over 6 months. Unfortunately, 8 months following her discharge, she presented with a deterioration in her respiratory status during her regular follow-up appointment. She reported experiencing an increase in breathlessness and was found to have a reduction in her oxygen saturation that decreased to 88% in the room air. On further clarification, it was uncovered that she had started taking oral escitalopram 10 mg once daily again. She received a prescription for this medication from a private psychiatry clinic that was not aware of her previous history. A chest X-ray captured at this stage revealed a worsening of the interstitial pattern that was consistent with a flare-up of sarcoidosis. Consequently, she was advised to discontinue the use of escitalopram, and the dosage of oral prednisolone was again increased to 50 mg once daily; as a result, she did not require readmission. An examination during a follow-up visit after 2 weeks revealed good improvement clinically and radiologically. She was again advised to avoid all SSRI medications; however, she was not satisfied with the effect of amitriptyline in controlling her depression.

Following this visit, she requested a full written report as she was moving to another city, and she did not attend anymore of her follow-ups.

## 3. Discussion

The relationship between SSRI and ILD has been previously described in the literature [14]; however, this case report demonstrates a possible association between PS and the use of the aforementioned SSRIs. At the time of her initial presentation, this patient was using a combination of two SSRIs for several months. She exhibited a poor response to therapeutic doses of systemic steroids while she was still being administered an SSRI, whereas she displayed an excellent response when the SSRI was discontinued. The observations during her follow-up, i.e., the development of a flare-up of PS in response to taking an SSRI again, also pointed to a clear correlation between this condition and taking SSRIs.

To our belief, these facts demonstrate the association between sarcoidosis and the use of more than the average recommended dose of SSRIs. This report should alert healthcare workers to this possible relationship and should stress the importance of excluding SSRI medications from a patient's treatment plan and of avoiding them in PS cases. In fact, other published case reports further demonstrate the relationship between SSRIs and other ILDs, and these data should affirm that the use of such medications in ILD cases is a real concern [14].

Rosenberg et al. [14] stated a possible mechanism that may induce lung involvement due to these medications. One of these mechanisms that may induce lung injury is by a nonspecific direct cytotoxic mechanisms and oxidative damage to alveoli, bronchioles, and capillaries in addition to immune mediation. Fluoxetine, which is one of these medications, has been shown to cause ILD in an animal model [13]. Genetic factors also had been claimed to be an important factor in inducing these reactions. These genetic factors may include polymorphisms in MUC5B promoter and cytochrome P450 [15].

Other suspected medications associated with drug-induced sarcoidosis have been overviewed by Cohen Aubert et al. [16]. This important overview included other medications like TNF-alpha antagonists, interferon or peg-interferon therapeutics, immune checkpoint inhibitors and some of pulmonary hypertension drugs.

In conclusion, drug-induced sarcoidosis is well documented in the literature, and this case report demonstrates that SSRI medications are a strong potential cause for PS, particularly when more than the average recommended dose or when combinations of more than one SSRI are used. Therefore, additional and meticulous precautions should be taken when SSRIs are used to treat patients with granulomatous lung diseases and ILDs.
